# Sex Differences in Speed‐Related Running Mechanics Using IMU in Runners

**DOI:** 10.1155/tsm2/6892376

**Published:** 2026-08-25

**Authors:** Raúl Cejudo-Alba, Xantal Borràs-Boix, Juan Pardo Albiach, Javier Martínez-Gramage

**Affiliations:** ^1^ University of Vic - Universitat Central de Catalunya, Vic, Spain; ^2^ Sport Exercise and Human Movement Research Group, Universitat de Vic - Universitat Central de Catalunya, Vic, Spain; ^3^ Department of Mathematics, Physics and Technology, Universidad Cardenal Herrera-CEU, CEU Universities, San Bartolomé 55, 46115, Alfara de Patriarca, Valencia, Spain, uoh.edu.sa; ^4^ Cathedra Villarreal CF-UCHCEU, Department of Physiotherapy, Faculty of Health Sciences, Universidad Cardenal Herrera - CEU, CEU Universities, Valencia, Spain

**Keywords:** braking-related deceleration, inertial measurement units (IMUs), runners, running biomechanics, sex differences

## Abstract

**Objective:**

The aim of this descriptive study was to evaluate lower‐limb biomechanical parameters in runners of different sexes and analyse how these parameters are modulated across three intensities (65%, 85% and 100% maximal aerobic speed [MAS]).

**Methods:**

One hundred recreational runners (50 women, 50 men) completed a 2000‐m time trial to determine MAS, followed by three 2‐min treadmill bouts at 65%, 85% and 100% MAS. Kinematic and mechanical variables were recorded using the RunScribe™ inertial measurement system, with sensors positioned bilaterally on the shoes and on the sacral region.

**Results:**

Linear mixed‐effects models showed a significant main effect of speed condition for most biomechanical outcomes. As speed increased from 65% to 100% MAS, runners showed shorter contact time and greater flight time, stride length, step rate, braking Gs, peak vertical ground reaction force, loading rates, shock, total shock and max pronation velocity. A significant main effect of sex was observed for several variables: men showed higher BG, flight time, stride angle, stride length, peak vertical ground reaction force, leg spring stiffness, loading rates and max pronation velocity, whereas women showed longer contact time. Sex × speed interactions were significant only for shock, total shock and max pronation velocity, indicating that most speed‐related adaptations occurred in a similar direction in men and women.

**Conclusions:**

These findings provide a descriptive characterisation of sex‐ and speed‐related IMU‐derived running mechanics in runners. Standardised treadmill testing across relative intensities may help characterise how lower‐limb mechanics are modulated by sex and running speed. Because injury incidence, running economy, race performance and longitudinal adaptations were not assessed, braking‐ and impact‐related variables should be interpreted as hypothesis‐generating rather than as evidence of injury risk or performance.

**Highlights**
• Sex and running speed effects on lower‐limb biomechanics were assessed using IMUs at 65%, 85% and 100% of MAS.
• As running speed increased, both sexes showed reduced contact time, greater stride length and increased braking Gs.
• The step rate was similar between sexes, whereas men showed greater stride length and stride angle and higher vertical and braking‐related acceleration metrics.

## 1. Introduction

Running is one of the most widely practised sporting activities worldwide, with over 107.9 million participants and approximately 70,000 organised events held annually [1]. Despite its well‐established health benefits and accessibility [2], running is associated with a substantial risk of injury. Nearly half of all runners sustain an overuse injury each year, with more injuries affecting the lower limbs (LLs) [3]. This considerable injury burden provides epidemiological context for research on running biomechanics; however, associations between individual biomechanical variables and injury cannot be inferred without prospective injury data. Moreover, biomechanical analysis is considered a key element in understanding performance. Running biomechanical analysis has been considered a key determinant of running economy (RE) [4] and has focused on contact time (CT) and spatiotemporal parameters [5]. In contrast, other wearable‐derived metrics such as braking Gs (BG) have received little attention in the scientific literature to date [6]. Recent literature indicates that recreational runners exhibit biomechanical patterns that may differ from those of elite runners, including less coordinated horizontal and vertical force production and lower RE [7]. In contrast to elite runners, recreational runners may rely more on proximal musculature during propulsion and less on ankle extensors, which has been associated with a less efficient running technique [8]. Collectively, this evidence highlights the need for descriptive data examining how men and women express speed‐related running mechanics when intensity is standardised relative to individual physiological capacity. Although several biomechanical adaptations to increasing running speed (RS) are already well established, fewer studies have characterised IMU‐derived running mechanics at high relative running intensities in a sex‐balanced sample.

Instrumented treadmills (TMs) have traditionally been a standard tool in biomechanical research [9] and have also been increasingly integrated into clinical settings to facilitate standardised assessments of running biomechanics [10]. A key distinction between TM and outdoor running is the absence of air resistance on the TM, which may compromise its ability to fully replicate natural running mechanics. On a TM, the runner does not move relative to the surrounding air, whereas in outdoor running, the body must overcome air resistance, which increases with RS [11]. Additionally, the centre‐of‐mass speed relative to the surrounding environment is effectively null, as movement results from the motion of the TM belt beneath the feet. To mitigate these mechanical discrepancies, a 1% incline has been proposed. After an appropriate familiarisation period, TM running does not significantly alter habitual running technique [12]. Reviews suggest that, although TM running does not fully replicate outdoor running, a 1% incline results in an energetic cost that more closely reflects real‐world conditions [9].

In parallel, inertial measurement units (IMUs) have become established as valid tools for biomechanical assessment in both laboratory and field settings. While IMUs typically include accelerometers, gyroscopes and magnetometers, the most common configuration employs only the first two sensors, as magnetometers are used less frequently [13]. Multiple studies have validated the use of IMUs against reference systems, demonstrating high accuracy in detecting gait events and estimating spatiotemporal and kinematic variables such as stride length (SL), step rate (SR) and CT, as well as in quantifying sagittal‐plane ankle kinematics [14]. A systematic review concluded that their validity ranges from moderate to excellent for these variables, although greater caution is advised for hip and knee measurements due to increased variability [14]. IMUs have proven effective in controlled laboratory environments [14–17] as well as in real‐world contexts, including long‐distance events such as marathons [18]. More sophisticated techniques, such as three‐dimensional (3D) motion capture and force platforms, offer greater precision but are limited by high cost, technical complexity and reduced ecological validity in replicating real‐world running conditions [9].

The aim of this descriptive study was to evaluate LL biomechanical parameters in runners of different sexes and analyse how these parameters are modulated across three intensities (65%, 85% and 100% maximal aerobic speed [MAS]).

## 2. Methods

### 2.1. Study Design, Ethical Approval and Participants

This cross‐sectional, laboratory‐based descriptive study with repeated measures was designed to characterise sex‐ and speed‐related differences in IMU‐derived LL running mechanics across three relative running intensities: 65%, 85% and 100% of MAS. The study protocol was reviewed and approved by the Research Ethics Committee of the Universitat de Vic—Universitat Central de Catalunya (CER UVic‐UCC; Approval No. 296/2023) on 6 November 2023. The study was conducted in accordance with the Declaration of Helsinki [19]. All participants provided written informed consent before participation.

The sample size was determined via prior analysis using G∗Power 3.1 software [20]. The calculation was based on an independent samples *t*‐test (men versus women), assuming a medium effect size, a statistical power of 80% and an alpha level of 0.05, indicating that a minimum of 100 participants were required. One hundred runners voluntarily participated in this study and provided written informed consent prior to participation.

Inclusion criteria were as follows: level of running exposure with ≥ 90 min per week across ≥ 2 sessions; age between 15 and 65 years; and absence of significant medical conditions, LL injuries within the previous year or surgeries that might affect biomechanical outcomes. Exclusion criteria included barefoot runners, use of minimalist or carbon‐plated footwear or foot orthoses, as well as current pregnancy. These criteria were used only to determine the eligibility and were not analysed as study variables. Participants were recruited via convenience sampling via social media, institutional outlets and distribution of informational materials at university facilities, sports clubs and local sports centres.

### 2.2. Experimental Protocol

This study employed a cross‐sectional, descriptive design conducted over two sessions. During the preparation, study procedures were explained, written informed consent was obtained, inclusion and exclusion criteria were verified, and baseline characteristics were recorded. Specifically, age was recorded, whereas body mass and height were measured. Participants were instructed to maintain their regular diet and training routine during the 48 h prior to testing and to avoid caffeine, alcohol and vigorous physical activity in the 24 h preceding the session.

The first session was oriented towards determining each participant’s MAS [21], took place on a certified 400‐m athletics track and consisted of a 2000‐m time trial.

Two weeks later, the biomechanical analysis was performed. This laboratory session lasted approximately 55 min. All testing was conducted in the same laboratory during morning and midday hours under controlled environmental conditions (20°C–22°C) to minimise potential circadian influences. Subjects used their habitual running footwear, and the same pair of shoes had to be used in both testing sessions.

Data were collected using IMUs (RunScribe™ system, Scribe Labs Inc., San Francisco, CA, USA). The system incorporates a 3‐axis gyroscope, 3‐axis accelerometer and 3‐axis magnetometer, with a sampling rate of 500 Hz. RunScribe™ validation and running‐gait studies have previously been reported [6, 22, 23]. The system was verified and calibrated according to the manufacturer’s guidelines (Supporting Information Table S1). No external synchronisation system was used. Participants were instrumented with IMUs following standardised hygiene protocols. Two pods were attached to the shoelaces of the left and right shoes, and a third sensor was positioned over the sacral region using a waistband. To minimise sensor movement, the manufacturer’s positioning and attachment instructions were followed.

Following instrumentation, participants completed a 10‐min running familiarisation on a TM (Medisoft RAM MAT 770S, Padova, Italy), which also served as the warm‐up period and consisted of progressively increasing running intensity. The TM incline was set at 1% throughout the warm‐up and all subsequent running bouts. RS was self‐selected to allow participants to adopt a comfortable pace before the experimental bouts. After warm‐up, participants performed three 2‐min bouts at 65%, 85% and 100% of their individual MAS. A 6‐min passive standing recovery period was provided between bouts to facilitate energy resynthesis and minimise fatigue accumulation. Throughout testing, participants were instructed to maintain a natural posture and their habitual running technique. All sessions were supervised by the same researcher to ensure protocol consistency and adherence to the manufacturer’s recommendations.

### 2.3. IMU‐Derived Biomechanical Variables

Data were transferred to the system dashboard and treated as step‐level outputs of the commercial system. No custom signal‐processing pipeline was implemented by the researchers. System processing algorithms were used to compute per‐stride summary metrics describing spatiotemporal parameters, segment kinematics and impact‐ and braking‐related acceleration metrics. Bilateral foot data were used to characterise LL mechanics, while sacral accelerations were used to characterise pelvis motion across running intensities. To avoid ambiguity and ensure consistent reporting, each variable is presented as variable name (abbreviation, unit).

The following variables were analysed: BG, g; CT, ms; flight time (FT), ms; horizontal ground reaction force rate (HGRF_R), N·kg^−1^·s^−1^; leg spring stiffness (LSS), kN·m^−1^; maximum pronation velocity (MPV), °·s^−1^; pronation excursion (PE), °; peak vertical ground reaction force (PVGRF), × BW; shock (SH), g; stride angle (SA), °; SL, m; SR, steps·min^−1^; swing force rate (SF_R), N·kg^−1^·s^−1^; time (T), ms; total shock (TS), g; RS, km·h^−1^; vertical ground reaction force rate (VGRF_R), N·kg^−1^·s^−1^; sacral BG (S_BG), g; sacral impact Gs (S_IG), g; sacral mediolateral Gs (S_MLG), g; and sacral vertical oscillation (S_VOSC), cm. Supporting Information Table S2 provides the operational definitions, brief narrative descriptions and units of the IMU‐derived variables included in the study.

### 2.4. Reliability Assessment of IMU‐Derived Variables

To assess intrasubject reliability of the IMU‐derived running biomechanics variables, a test–retest design was previously implemented. A subgroup of 20 runners (10 women and 10 men) completed two identical laboratory sessions separated by 2–7 days. In both sessions, after a standardised TM warm‐up, participants performed three repeated 3‐min running bouts at 85% of their individual MAS, using the same TM conditions, instruments and sensor placement, and procedural instructions as in the main experimental protocol.

For each running bout, the first and last 30 s were excluded, and the central 2 min was retained to focus on a stable running pattern and to minimise the influence of initial acceleration and end‐of‐bout fatigue. For each participant and session, values obtained from the three repeated bouts were averaged before calculating test–retest reliability. Therefore, reliability analyses were conducted at the participant level, using one mean test value and one mean retest value per participant for each IMU‐derived variable. Intersession consistency was evaluated using the intraclass correlation coefficient (ICC), while the standard error of measurement (SEM), SEM expressed as a percentage of the mean (SEM%) and coefficient of variation (CV%) were calculated as indices of absolute reliability. ICC values were obtained using SPSS, based on a two‐way mixed‐effects model, absolute agreement and single measurements. SEM was calculated as SD × √(1 − ICC), SEM% as (SEM/mean) × 100 and CV% as (within‐subject SD/mean) × 100, with within‐subject SD estimated as the standard deviation of the paired test–retest differences divided by √2.

Table S3 presents the test–retest reliability results for the IMU‐derived running biomechanics variables assessed at 85% of MAS, including ICC, 95% confidence intervals, SEM, SEM%, CV%, minimum detectable change at the 95% confidence level and qualitative reliability classification.

### 2.5. Statistical Analysis

Upon completion of the biomechanical assessment procedures for all participants, the data were stored in a custom‐designed database specific to this study. A subsequent data cleansing process was conducted, including the detection of potential recording errors and the identification of outliers due to monitoring data systems. Following data cleansing, descriptive and inferential statistical analyses were performed using various libraries within the RStudio environment [24]. To address the repeated‐measures structure of the data and reduce the reliance on multiple univariate comparisons, linear mixed‐effects models were fitted for the main biomechanical outcomes. For each dependent variable, sex, RS condition and the sex × speed interaction were included as fixed effects, while participant ID was included as a random intercept to account for within‐subject correlation across the three speed conditions.

Type III ANOVA tables were obtained from each linear mixed‐effects model to test the main effects of sex and speed condition, as well as the sex × speed interaction, while accounting for repeated measurements within participants. The complete Type III ANOVA outputs for all biomechanical outcomes are provided in the supporting Excel spreadsheet ‘IMU_Linear_Mixed_Effects_Model_Results.xlsx’. To reduce the risk of inflated Type I error due to multiple testing across biomechanical outcomes, *p* values obtained from the Type III ANOVA tables were adjusted using the Benjamini–Hochberg false discovery rate (FDR) procedure. Adjusted *p* values are reported as p_FDR. When significant main effects or interactions were identified, post hoc pairwise comparisons of estimated marginal means were made to examine differences between sexes within each speed condition and differences between speed conditions within each sex. These post hoc comparisons were adjusted for multiple comparisons using the Holm procedure within each family of comparisons.

Effect estimates were reported with 95% confidence intervals. Partial eta‐squared effect sizes were calculated to quantify the magnitude of the fixed effects, and marginal and conditional *R* ^2^ values were used to assess the model fit. Marginal *R* ^2^ represented the proportion of variance explained by the fixed effects, whereas conditional *R* ^2^ represented the proportion of variance explained by both fixed and random effects.

## 3. Results

### 3.1. Participants and Descriptive Characteristics

Descriptive characteristics of the sample are presented in Table 1. The sample comprised 100 runners, 50 men and 50 women. The total sample had a mean body mass of 64.0 ± 10.1 kg, height of 170.0 ± 8.7 cm, age of 33.6 ± 9.6 years and MAS of 15.0 ± 2.1 km·h^−1^. Men displayed significantly higher values for height, body mass and MAS than women (*p* < 0.0001), whereas no significant differences were observed in age between sexes (*p* = 0.3102). Most participants reported running at least 3 days per week (85%); weekly running frequency was descriptive only and was not included in the inferential models.

**TABLE 1:** Descriptive characteristics of runners, stratified by sex (*n* = 100; 50 men and 50 women).

Variable (unit)	Sex		Total
	Male	Female	
Body mass (kg)	70.7 (8.9)	57.4 (5.9)	64.0 (10.1)
Height (cm)	176.1 (6.8)	163.9 (5.6)	170.0 (8.7)
Age (years)	34.3 (10.4)	33.0 (8.8)	33.6 (9.6)
MAS (km·h^−1^)	16.2 (1.8)	13.8 (1.7)	15.0 (2.1)
Weekly RF (days/week)	3.0 (0.1)	2.9 (0.3)	2.9 (0.3)

*Note:* Values are presented as mean (SD). Weekly RF was collected categorically (1–2, 2–3 or > 3 days/week), presented for sample description only, and not included in the inferential models. cm, centimetre; kg, kilogram; km·h^−1^, kilometres per hour.

Abbreviations: MAS, maximal aerobic speed; SD, standard deviation; weekly RF, weekly running frequency.

### 3.2. Linear Mixed‐Effects Model Overview

Linear mixed‐effects models were fitted for each biomechanical outcome to account for the repeated‐measures structure of the data. Sex, speed condition and the sex × speed interaction were included as fixed effects, whereas participant ID was included as a random intercept. Each model included 300 observations from 100 participants, corresponding to the three relative speed conditions assessed in each runner. No singular fit was detected in any of the fitted models. Overall, the mixed‐effects models showed that RS was the main determinant of variation in IMU‐derived running mechanics. Significant main effects of the speed condition were observed for most biomechanical variables, including BG, CT, FT, T, SA, SL, SR, PVGRF, VGRF_R, HGRF_R, SF_R, SH, TS, S_IG, PE, MPV, S_MLG, S_VOSC and S_BG (all p_FDR < 0.001).

### 3.3. Speed‐Related Changes in IMU‐Derived Running Mechanics

Descriptive values for all IMU‐derived biomechanical variables stratified by sex and speed condition are presented in Table 2. Consistent with these descriptive data, as RS increased from 65% to 100% of MAS, runners showed systematic changes in spatiotemporal, kinematic and mechanical loading variables. Specifically, CT and T decreased progressively with increasing speed, whereas FT, SL, SR, BG, PVGRF, VGRF_R, HGRF_R, SF_R, SH, TS and MPV increased. These findings indicate that increasing relative running intensity produced consistent speed‐dependent modifications in running mechanics and mechanical loading.

**TABLE 2:** Descriptive IMU‐derived biomechanical outcomes stratified by sex and running speed condition (*n* = 100).

Variable (unit)	Male			Female		
	Threshold 65%	Threshold 85%	Threshold 100%	Threshold 65%	Threshold 85%	Threshold 100%
Braking Gs (g)	8.58 (2.18)	9.12 (2.22)	9.62 (2.12)	7.38 (2.26)	8.01 (2.00)	8.61 (2.11)
Contact time (ms)	282.48 (33.12)	244.00 (24.56)	225.16 (22.61)	299.38 (38.94)	257.92 (26.97)	237.40 (25.32)
Flight time (ms)	77.66 (32.32)	98.16 (25.77)	107.42 (27.18)	63.14 (33.69)	83.02 (19.66)	85.82 (18.61)
Horizontal GRF rate (N·kg^−1^·s^−1^)	5.86 (1.99)	7.61 (1.76)	8.71 (1.54)	5.18 (1.13)	7.25 (1.60)	8.39 (1.70)
Leg spring stiffness (kN·m^−1^)	9.50 (1.93)	9.78 (1.81)	10.07 (1.78)	8.06 (1.49)	7.76 (1.42)	8.16 (1.91)
Max pronation velocity (°·s^−1^)	606.14 (163.77)	749.56 (190.26)	904.62 (232.21)	505.26 (138.01)	632.68 (180.37)	732.26 (181.83)
Pronation excursion (°)	−13.38 (5.36)	−15.43 (6.17)	−16.08 (7.34)	−13.04 (4.18)	−14.65 (4.53)	−15.15 (4.48)
Peak vertical GRF (× BW)	3.15 (0.51)	3.68 (0.53)	3.91 (0.56)	2.90 (0.55)	3.33 (0.42)	3.50 (0.41)
Shock (g)	12.05 (2.33)	13.44 (2.39)	14.06 (2.32)	11.30 (2.25)	12.97 (2.12)	14.23 (2.05)
Stride angle (°)	1.93 (1.15)	2.26 (0.83)	2.23 (0.74)	1.50 (0.88)	1.81 (0.67)	1.80 (0.54)
Stride length (m)	2.05 (0.42)	2.52 (0.42)	2.75 (0.40)	1.73 (0.34)	2.23 (0.42)	2.46 (0.52)
Step rate (steps·min^−1^)	167.44 (8.65)	176.46 (9.93)	184.68 (10.96)	167.72 (8.21)	176.76 (7.83)	186.08 (9.66)
Swing force rate (N·kg^−1^·s^−1^)	1.17 (0.30)	1.46 (0.30)	1.70 (0.30)	0.96 (0.20)	1.33 (0.31)	1.61 (0.44)
Time (MP‐> TO) (ms)	221.38 (37.18)	184.98 (24.28)	170.40 (21.78)	237.04 (38.03)	197.24 (23.51)	180.30 (22.61)
Total shock (g)	4091.10 (867.92)	4794.60 (992.05)	5245.68 (1038.74)	3804.56 (823.11)	4602.98 (859.84)	5304.82 (892.13)
Running speed (km·h^−1^)	10.53 (1.19)	13.8 (1.6)	16.2 (1.9)	8.95 (1.08)	11.7 (1.4)	13.7 (1.7)
Vertical GRF rate (N·kg^−1^·s^−1^)	35.24 (3.79)	40.72 (3.86)	44.01 (4.44)	33.34 (3.99)	38.50 (3.97)	41.83 (4.17)
Sacral braking Gs (g)	2.76 (1.44)	3.38 (1.68)	3.78 (1.75)	2.88 (1.62)	3.23 (1.62)	3.58 (1.61)
Sacral impact Gs (g)	4.64 (2.29)	4.75 (2.26)	4.82 (2.11)	5.35 (2.37)	5.56 (2.28)	5.61 (2.27)
Sacral mediolateral Gs (g)	2.38 (0.97)	2.98 (1.35)	3.11 (1.30)	2.65 (1.04)	2.98 (1.13)	3.27 (1.20)
Sacral vertical oscillation (cm)	8.11 (1.60)	7.09 (1.66)	6.12 (1.61)	8.25 (1.47)	6.97 (1.70)	5.60 (1.19)

*Note:* Values are presented as mean (SD). Table 2 is descriptive only. Accordingly, no significance symbols are displayed. Inferential comparisons between running speed conditions within each sex and between sexes within each speed condition were obtained from the linear mixed‐effects models. Exact *p* values, globally FDR‐adjusted *p* values and 95% confidence intervals for these comparisons are reported in Additional File 1. Abbreviations and operational definitions are provided in Supporting Information Table S4.

CT decreased progressively with speed in both sexes. In women, CT decreased from 299.38 ms at 65% MAS to 257.92 ms at 85% MAS and 237.40 ms at 100% MAS. In men, CT decreased from 282.48 ms to 244.00 ms and 225.16 ms, respectively. Women showed significantly longer CT than men at 65% MAS and 85% MAS, whereas the difference at 100% MAS did not remain significant after global FDR adjustment.

FT increased with speed and was consistently higher in men. In women, it increased from 63.14 ms at 65% MAS to 83.02 ms at 85% MAS and 85.82 ms at 100% MAS, whereas in men, it increased from 77.66 ms to 98.16 ms and 107.42 ms, respectively. Sex differences were significant at all three speed conditions.

SL increased progressively with speed in both sexes and was consistently greater in men. In women, it increased from 1.73 m at 65% MAS to 2.23 m at 85% MAS and 2.46 m at 100% MAS, whereas in men, it increased from 2.05 m to 2.52 m and 2.75 m, respectively. Men showed significantly longer SL than women at all speed conditions.

SR also increased significantly with speed in both sexes, but no significant sex differences were observed. Women showed estimated values of 167.72, 176.76 and 186.08 steps·min^−1^ at 65%, 85% and 100% MAS, respectively, while men showed values of 167.44, 176.46 and 184.68 steps·min^−1^. Therefore, in both sexes, SR increased with speed, but sex‐related differences were more evident in SL and loading‐related variables than in SR.

**FIGURE 1:**
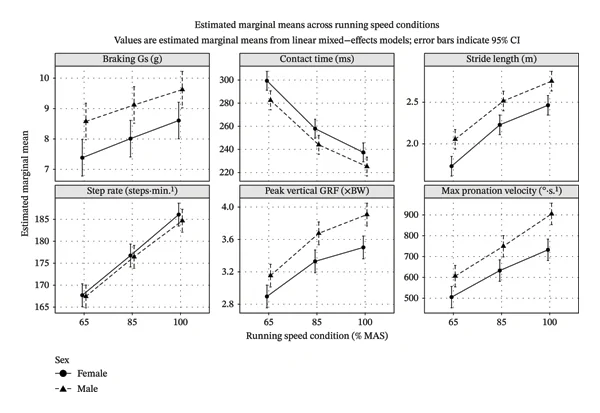
Estimated marginal means for key biomechanical outcomes across relative running speed conditions. Values are estimated marginal means from the linear mixed‐effects models; error bars indicate 95% confidence intervals. BG, braking Gs; CI, confidence interval; CT, contact time; MAS, maximal aerobic speed; MPV, maximum pronation velocity; PVGRF, peak vertical ground reaction force; SL, stride length; SR, step rate.

These speed‐related patterns are visually summarised in Figure 1, which presents the estimated marginal means and 95% confidence intervals for selected key biomechanical outcomes. The figure illustrates the progressive decrease in CT and the increases in BG, SL, SR, PVGRF and MPV as relative RS increased in both sexes.

### 3.4. Sex‐Related Differences in Running Mechanics

A significant main effect of sex was observed for 11 biomechanical variables. Men showed higher values than women for BG, FT, SA, SL, PVGRF, LSS, VGRF_R, SF_R and MPV. Conversely, women showed longer CT and T. These results indicate sex‐related differences in selected IMU‐derived running mechanical outcomes, independently of the speed condition.

PVGRF increased with speed and was consistently higher in men. Women showed estimated values of 2.90 × BW at 65% MAS, 3.33 × BW at 85% MAS and 3.50 × BW at 100% MAS. Men showed values of 3.15 × BW, 3.68 × BW and 3.91 × BW, respectively. Sex differences were significant at 65%, 85% and 100% MAS. Similar speed‐dependent increases were observed for VGRF_R and HGRF_R.

LSS showed a significant sex effect but no significant speed effect. Men presented higher LSS than women at all speed conditions.

### 3.5. Sex × Speed Interaction Effects

The sex × speed interaction was significant only for SH, TS and MPV. No significant sex × speed interaction was observed for BG, CT, SL, SR, PVGRF, or most other variables. Therefore, although men and women differed in several biomechanical outcomes, the general pattern of adaptation to increasing speed was broadly similar between sexes for most variables.

MPV increased markedly with speed and was higher in men at all speed conditions. Women showed estimated values of 505.26°·s^−1^ at 65% MAS, 632.68°·s^−1^ at 85% MAS and 732.26°·s^−1^ at 100% MAS. Men showed values of 606.14, 749.56 and 904.62°·s^−1^, respectively. The significant sex × speed interaction for MPV indicates that the increase in MPV with RS was greater in men than in women, particularly at the highest intensity.

SH and TS increased significantly with speed in both sexes. For women, SH increased from 11.30 g at 65% MAS to 12.97 g at 85% MAS and 14.23 g at 100% MAS, whereas for men, SH increased from 12.05 g to 13.44 g and 14.06 g, respectively. For women, TS increased from 3804.56 to 4602.98 and 5304.82, whereas for men, TS increased from 4091.10 to 4794.60 and 5245.68. The significant sex × speed interactions for SH and TS indicate that the speed‐related progression of these variables was not identical between sexes.

Estimated marginal means showed that BG increased progressively with speed in both sexes. In women, BG increased from 7.38 g at 65% MAS to 8.01 g at 85% MAS and 8.61 g at 100% MAS. In men, BG increased from 8.58 g at 65% MAS to 9.12 g at 85% MAS and 9.62 g at 100% MAS. Post hoc comparisons showed significantly higher BG in men than in women at 65% MAS, 85% MAS and 100% MAS. The mean difference between women and men was −1.19 g at 65% MAS, −1.11 g at 85% MAS and −1.02 g at 100% MAS. Within‐sex comparisons showed that BG increased significantly between all speed conditions in both women and men.

However, the sex × speed interaction was not significant for BG. This indicates that although men showed higher BG values overall, the speed‐related increase in BG followed a broadly similar pattern in both sexes.

### 3.6. Effect Sizes and Model‐Explained Variance

**FIGURE 2:**
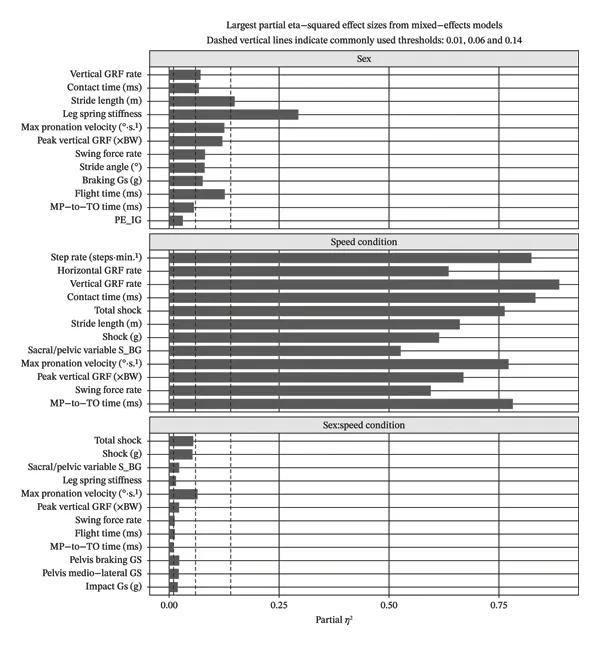
Largest partial eta‐squared effect sizes from the linear mixed‐effects models for sex, speed condition and the sex × speed interaction. The dashed vertical lines indicate commonly used thresholds for small (0.01), medium (0.06) and large (0.14) effects. MPV, maximum pronation velocity; PVGRF, peak vertical ground reaction force; SH, shock; TS, total shock.

Effect size analysis showed that the speed condition had large effects on several variables, particularly VGRF_R, CT, SR, T, MPV, TS, PVGRF, SL, HGRF_R and SH. The largest sex‐related effects were observed for LSS, SL, FT, MPV and PVGRF. Interaction effect sizes were smaller and mainly observed for MPV, TS and SH, consistent with the limited number of significant sex × speed interactions observed in the mixed‐effects models. These effect size patterns are visually summarised in Figure 2, which presents the largest partial eta‐squared values for the effects of the speed condition, sex and the sex × speed interaction across the biomechanical outcomes.

Conditional *R* ^2^ values were substantially higher than marginal *R* ^2^ values for several outcomes, indicating that participant‐specific variability accounted for an important proportion of the total variance. These results support the use of mixed‐effects models and indicate that, although sex and speed explained a relevant proportion of biomechanical variability, individual runner characteristics also contributed substantially to the observed responses.

Complete linear mixed‐effects model outputs, estimated marginal means, post hoc comparisons, exact *p* values, globally FDR‐adjusted *p* values, 95% confidence intervals, effect sizes and marginal and conditional *R* ^2^ values are provided in Additional File 1 (Microsoft Excel file).

## 4. Discussion

The present study examined whether sex and relative RS affect IMU‐derived LL biomechanical variables in runners assessed at three intensities based on individual MAS. The mixed‐effects analysis showed that relative RS was the principal factor associated with changes in running mechanics, with sex‐related differences observed in selected biomechanical variables. Accordingly, the findings should be interpreted as a descriptive characterisation of IMU‐derived running mechanics under standardised relative intensity conditions, rather than as evidence of distinct sex‐specific braking strategies or clinically relevant loading profiles.

### 4.1. Relative Intensity and Speed‐Related Mechanical Demand

The present protocol adds a relative intensity perspective to this evidence. Participants were assessed at equivalent percentages of their individual MAS rather than at identical absolute TM speeds. Therefore, the study describes how IMU‐derived mechanical variables are expressed under comparable relative demands, while acknowledging that men and women were not necessarily running at the same external velocity within each condition.

Sex‐related differences in ground reaction force patterns have been reported as RS increases [25]. Changes in RS have also been shown to modify the waveform structure of ground reaction forces, indicating that speed‐related mechanical changes extend beyond isolated peak values [26]. These findings provide a kinetic context for interpreting the present results, but they do not constitute direct confirmation because the current study examined acceleration‐derived wearable outcomes rather than direct force measurements.

Cadence has been shown to be influenced by RS and leg length [27]. Therefore, the limited differentiation in SR between sexes should not be interpreted as indicating equivalent running mechanics. Instead, it suggests that temporal gait regulation may be closely linked to the imposed running demand, while spatial and acceleration‐derived outcomes may provide complementary information under relative intensity conditions.

### 4.2. Sex‐Related Mechanical Profiles Under Standardised Relative Intensity

Sex‐related differences in running biomechanics have previously been identified, particularly in LL joint kinematics [28]. The present study contributes to this literature through a different measurement domain by examining stride‐level and acceleration‐derived outcomes obtained from wearable sensors during running at standardised relative intensities.

The current differences should not be interpreted as effects of sex in isolation. Although relative intensity was standardised, men and women in the present sample differed in anthropometric characteristics and MAS. In addition, the mixed‐effects models indicated that participant‐specific variability contributed substantially to the mechanical responses. The findings are therefore best understood as sex‐related mechanical profiles expressed under the present testing conditions, rather than as fixed movement strategies attributable exclusively to sex.

Stride organisation provides a biomechanical framework for interpreting these profiles, as changes in stride frequency and foot position at landing have been associated with braking force and impact peak force, while broader alterations in stride organisation may also influence the metabolic cost of running [29]. These findings support considering spatial gait characteristics when interpreting wearable‐derived braking and impact signals. However, because landing position, direct braking force and metabolic cost were not assessed in the present study, the mechanisms underlying the observed sex‐related profiles cannot be determined.

### 4.3. Interpretation of IMU‐Derived Loading and Interaction Outcomes

Among the IMU‐derived outcomes, BG represents an acceleration‐derived indicator of braking‐related deceleration during stance. The updated analysis does not support a sex‐dependent response of BG to increasing relative speed. Accordingly, BG should be interpreted as a variable that is sensitive to relative RS and differs in overall magnitude between sexes, but not as evidence that men and women adopt distinct speed‐specific braking strategies.

The validity and reliability of IMU‐derived running outcomes vary according to the specific variable assessed and the sensor location used [14]. Measurement accuracy during high‐speed and maximal‐effort running also depends on the biomechanical outcome and testing condition [16]. Therefore, force platform findings are useful for contextualising the present results, but direct braking force measures and acceleration‐derived BG should not be considered mechanically interchangeable. Wearable acceleration features have previously been used to distinguish running pattern characteristics between male and female runners and between competitive and recreational runners [30].

### 4.4. Selective Interaction Effects and Measurement‐Specific Considerations

The interaction effects identified for SH, TS and MPV indicate that sex‐dependent responses to increasing relative speed were restricted to selected outcomes rather than generalised across the complete set of variables. Therefore, the results do not support the existence of broadly different sex‐specific responses to increasing relative intensity. Instead, these variables should be regarded as outcomes requiring further targeted investigation.

The interpretation of MPV requires particular caution because IMU‐derived running measurements vary in validity according to the specific outcome assessed [14]. Accuracy during high‐speed running may also vary according to the kinematic variable under examination [16]. Thus, the interaction involving MPV should be confirmed using independent motion analysis systems before being interpreted as evidence of different foot motion control between sexes.

A similarly cautious interpretation is appropriate for pelvic‐related outputs obtained from the sacral configuration. Validation research using the RunScribe Sacral Gait Lab™ system reported that the device did not reach the established validity criteria for pelvic kinematic variables across the walking and RSs evaluated [22]. Accordingly, pelvic kinematic outcomes derived from the sacral sensor should be interpreted descriptively within the measurement boundaries of the commercial system.

### 4.5. Methodological Contribution and Transferability

A methodological strength of the present study is the combination of a sex‐balanced sample with a running intensity protocol normalised to individual MAS. This approach permits the comparison of IMU‐derived mechanics under equivalent relative intensity conditions and avoids relying only on comparisons at identical absolute velocities between groups with different aerobic capacities.

Motorised TM running and overground running are broadly comparable for several biomechanical outcomes, although they are not identical conditions [9]. Specific kinematic and kinetic differences have also been reported between TM and overground running [12]. Thus, the present findings should be interpreted as evidence obtained under controlled TM conditions rather than as a direct representation of outdoor running mechanics.

A 1% incline has been shown to approximate the energetic cost of outdoor running more accurately than level TM running within a defined range of speeds [31]. However, similarity in energetic demand does not establish complete biomechanical equivalence between TM and overground running.

Wearable‐sensor monitoring has identified changes in running mechanics during continuous marathon running [18]. Prolonged running has also been shown to modify determinants of endurance performance after 90 min, with these changes becoming more pronounced after 120 min [32]. Consequently, the mechanical profiles described in the present study should be understood as acute responses under controlled laboratory conditions rather than as representative patterns of prolonged or fatigued running.

### 4.6. Implications for Wearable‐Based Running Assessment

The practical contribution of the present study lies in the descriptive use of wearable sensors to characterise individual mechanical responses across standardised relative running intensities. This approach may be useful for identifying variables that deserve further examination under controlled and field‐based conditions. However, the present findings do not support the use of BG, SH, TS or MPV as isolated criteria for clinical decision‐making, injury‐prevention strategies or performance prescription, because these outcomes were not assessed in the study design.

The substantial contribution of participant‐specific variability indicates that subsequent wearable‐based analyses should consider factors beyond sex and RS. Future studies should examine anthropometric characteristics, running experience, training exposure, footwear and habitual technique; these factors were not analysed here.

Further research should combine IMU‐derived variables with independent kinetic and 3D kinematic reference systems, because the interpretation of wearable‐derived variables depends on outcome‐specific validation [14]. Continuous wearable monitoring during prolonged running has already been shown to be feasible in ecological conditions [18]. Combining laboratory‐based relative intensity testing with outdoor and fatigue‐related assessments will therefore be necessary to determine the broader relevance of the mechanical profiles described in the present study.

## 5. Limitations

First, the descriptive, cross‐sectional laboratory design may not reflect outdoor running biomechanics or the demands of prolonged running, limiting ecological validity. The three intensity conditions (65%, 85% and 100% MAS) were evaluated in standardised 2‐min bouts without accumulated fatigue; therefore, whether the observed patterns persist under longer and more demanding conditions remains unknown. In addition, although 100% MAS was included to characterise responses at a controlled high relative intensity, this condition may not reflect the habitual intensity or duration of typical running.

Second, participants were runners recruited via convenience sampling within a relatively restricted age range, which may limit generalisability. Weekly mileage, years of running experience and detailed injury history were not collected or analysed.

Third, biomechanical outcomes were derived from IMU‐based estimates relying on proprietary algorithms, and test–retest reliability was assessed only in a subgroup of participants (*n* = 20) at a single submaximal intensity (85% MAS). Therefore, these reliability results may not generalise to maximal‐speed running or to all relative intensity conditions assessed in the main protocol. In addition, reliability was not uniform across all variables. Although most IMU‐derived variables showed good‐to‐excellent test–retest reliability, PE showed poor reliability and high relative measurement error; therefore, findings involving this variable should be interpreted with caution.

Finally, menstrual cycle phase and hormonal contraceptive use were not recorded or controlled in women participants; therefore, potential cycle‐related influences on the assessed biomechanical outcomes could not be examined. Future studies should include standardised menstrual cycle monitoring and reporting procedures—and, where feasible, hormonal characterisation—to determine whether cycle status contributes to within‐women variability in running mechanics [33].

Collectively, these limitations highlight the need for longitudinal and field‐based studies with improved ecological validity, standardised participant characterisation and direct assessment of injury, RE and performance outcomes.

## 6. Conclusions

In the present study, sex‐ and speed‐related IMU‐derived running mechanics were characterised in runners assessed under standardised TM conditions at 65%, 85% and 100% of individual MAS. Relative RS was the main factor influencing LL running mechanics.

Sex‐related differences were identified in selected biomechanical outcomes, whereas sex × speed interactions were limited to SH, TS and MPV, indicating that most speed‐related responses occurred in a broadly similar direction in men and women.

BG increased with speed and was higher in men across speed conditions, but its speed‐related response did not show a significant sex‐dependent pattern. Therefore, BG should be interpreted as an IMU‐derived braking‐related acceleration variable rather than as evidence of distinct sex‐specific speed–braking strategies.

Overall, these findings provide descriptive reference data on sex‐ and speed‐related IMU‐derived running mechanics across relative intensities. Because injury outcomes were not measured, no conclusions regarding injury risk or injury prevention can be drawn from these findings; any potential relationship between the observed biomechanical patterns and injury should be considered hypothesis‐generating. Future longitudinal, field‐based and validation studies with prospectively measured injury outcomes are needed to clarify the broader applicability of these metrics beyond controlled TM assessment.

## Author Contributions

Raúl Cejudo‐Alba, Xantal Borràs‐Boix and Javier Martínez‐Gramage conceived the study; Raúl Cejudo‐Alba collected the data; Raúl Cejudo‐Alba, Javier Martínez‐Gramage and Juan Pardo Albiach analysed the data and wrote the initial manuscript draft; and Raúl Cejudo‐Alba, Xantal Borràs‐Boix, Javier Martínez‐Gramage and Juan Pardo Albiach contributed to the draft updates.

## Funding

No funding was received for this manuscript.

## Disclosure

All authors approved the final version.

## Ethics Statement

Ethical approval for this study was granted by the Research Ethics Committee of the Universitat de Vic—Universitat Central de Catalunya (CER UVic‐UCC; Approval No. 296/2023) on 2 December 2023. All study procedures complied with the Declaration of Helsinki.

## Consent

Prior to the start of the study, all participants were fully informed about the procedures, potential risks and benefits of the research. Written informed consent was obtained from each participant, who voluntarily agreed to take part in the study.

## Conflicts of Interest

The authors declare no conflicts of interest.

## Supporting Information

Additional supporting information can be found online in the Supporting Information section.

## Supporting information


**Supporting Information 1** Supporting Information Table S1 provides the manufacturer documentation sources consulted for RunScribe™ hardware specifications, dashboard‐derived metrics and validation information. Supporting Information Table S2 provides the operational definitions and units of the IMU‐derived variables obtained using RunScribe™. Supporting Information Table S3 presents the test–retest reliability results for the IMU‐derived running biomechanics variables assessed at 85% of MAS. Supporting Information Table S4 provides the complete alphabetical list of abbreviations and symbols used throughout the manuscript.

**Supporting Information 2** The supporting Excel spreadsheet ‘IMU_mixture_models_results_paper.xlsx’ provides the complete Type III ANOVA outputs for all IMU‐derived biomechanical outcomes.

## Data Availability

The data supporting the findings of this study are provided in the accompanying Supporting Information tables and in Additional File 1. The latter contains the complete results of the linear mixed‐effects analyses, including estimated marginal means, post hoc comparisons, exact *p* values, globally FDR‐adjusted *p* values, 95% confidence intervals, effect sizes and marginal and conditional *R* ^2^ values. Additional information may be obtained from the corresponding author upon reasonable request.
