# Supplementary material for: Sex Differences in Speed‐Related Running Mechanics Using IMU in Runners
Source: Transl Sports Med. 2026 Aug 25;2026:6892376. doi: 10.1155/tsm2/6892376 (PMC13505192; doi:10.1155/tsm2/6892376)
Supplement: Supplementary file 1 [file TSM2-2026-6892376-s002.docx]

**Supporting Information**

Additional supporting information can be found online in the Supporting Information section.

**Suplementary material**

**Table S1 |** Manufacturer documentation links consulted for RunScribe™ hardware specifications, dashboard-derived metrics, and validation information.

| **Source** | **Information used** | **Link** |
| --- | --- | --- |
| RunScribe™ hardware specifications | Sensor type and sampling frequency up to 500 Hz | [Link 1](https://runscribe.com/projects/) |
| RunScribe™ product description | Description of RunScribe™ as a gait-analysis platform | [Link 2](https://runscribe.com/) |
| RunScribe™ metrics documentation | Contact time, flight time, stride rate, Impact Gs, and Braking Gs | [Link 3](https://runscribe.com/metrics/) |
| RunScribe™ dashboard export example | Example of dashboard-derived metrics, including Braking Gs | [Link 4](https://www.move-support.nl/index_htm_files/RunScribe-353978.pdf) |
| RunScribe™ ground contact time validation report | Embedded footstep model and validation information | [Link 5](https://runscribe.com/runscribe-validation-research-study/) |

**Table S2 |** Operational definitions and units of the IMU-derived variables (Runscribe™).

| **Variable (abbr.)** | **Brief narrative definition** | **Unit** |
| --- | --- | --- |
| Braking Gs (BG) | Peak anteroposterior acceleration at ground contact (IMU-based proxy of braking/deceleration; acceleration, not force). | g |
| Contact time (CT) | Time the foot is in contact with the ground from footstrike to toe-off. | ms |
| Flight time (FT) | Time spent in aerial phase (no ground contact) within the stride. | ms |
| Horizontal GRF rate (HGRF_R) | Estimated mean horizontal force rate during the propulsive phase. | N·kg⁻¹·s⁻¹ |
| LEG SPRING STIFFNESS (LSS) | Estimated leg spring stiffness during running using a spring-mass approach. | kN·m⁻¹ |
| MAX PRONATION VELOCITY (MPV) | Peak angular velocity of pronation between footstrike and maximum pronation. | °·s⁻¹ |
| TIME (MP→TO) (T) | Time elapsed from mid-stance to toe-off within the stance phase (gait-event based). | ms |
| PEAK VERTICAL GRF (F(Bw)) (PVGRF) | Estimated peak vertical ground reaction force at/around mid-stance, normalized to body weight. | ×BW |
| SACRAL BRAKING GS (G) (S_BG) | Peak anteroposterior acceleration at ground contact measured at the sacrum. | g |
| SACRAL IMPACT GS(G) (S_IG) | Peak vertical acceleration at ground contact measured at the sacrum. | g |
| SACRAL MEDIO LATERAL GS (G) (S_MLG) | Peak medio-lateral acceleration at ground contact measured at the sacrum. | g |
| SACRAL VERTICAL OSCILLATION (S_VOSC) | Vertical displacement of the sacrum across the gait cycle (peak-to-peak, max–min). | cm |
| PRONATION EXCURSION (PE) | Total rearfoot pronation range of motion from footstrike to maximum pronation during ground contact. | ° |
| SHOCK (SH) | Proprietary composite metric integrating Impact Gs and Braking Gs into a single step-level “shock” indicator. | g |
| STEP RATE (SR) | Cadence; number of steps performed per minute. | steps·min⁻¹ |
| STRIDE ANGLE (SA) | Angle describing the parabolic trajectory of the stride, derived from stride length and foot rise during swing. | ° |
| STRIDE LENGTH (SL) | Distance between two consecutive placements of the same foot (i.e., two step lengths). | m |
| SWING FORCE RATE (S_FR) | Estimated mean rate of force required to swing the limb during the stride. | N·kg⁻¹·s⁻¹ |
| TOTAL SHOCK (TS) | Proprietary metric derived from Shock representing overall shock load as reported by the manufacturer. | Gs |
| RUNNING SPEED (RS) | Running speed during the treadmill bout. | m·s⁻¹ |
| VERTICAL GROUND REACTION FORCE RATE (VGRF_R) | Estimated mean vertical force rate during stance. | N·kg⁻¹·s⁻¹ |

**Table S3 |** Test–retest reliability of IMU-derived running biomechanics variables at 85% of maximal aerobic speed (MAS)

This supplementary file reports the test–retest reliability of biomechanical variables recorded with the RunScribe™ IMU system during treadmill running performed exclusively at 85% of maximal aerobic speed (MAS). Reliability is presented for each variable (and for each side/sensor location where applicable), using both relative and absolute indices to quantify the between-session stability of the measurements. Specifically, we report the intraclass correlation coefficient (ICC) with 95% confidence intervals, the standard error of measurement (SEM; SEM%), the coefficient of variation (CV%), and the minimum detectable change at the 95% confidence level (MDC95).

| **Variable** | **ICC** | **IC95%** | **Mean** | **Range** | **Variance** | **SD** | **SEM** | **SEM%** | **CV%** | **Category** |
| --- | --- | --- | --- | --- | --- | --- | --- | --- | --- | --- |
| Step Rate (/min) | 0.987 | 0.967–0.995 | 178.642 | 36.000 | 90.950 | 9.537 | 1.102 | 0.62% | 0.63% | Excellent |
| Stride Length (m) | 0.971 | 0.643–0.993 | 2.196 | 2.283 | 0.287 | 0.536 | 0.091 | 4.13% | 2.71% | Excellent |
| Contact Time (ms) | 0.975 | 0.917–0.991 | 256.117 | 89.333 | 508.061 | 22.540 | 3.543 | 1.38% | 1.21% | Excellent |
| Flight Time (ms) | 0.974 | 0.907–0.991 | 23.842 | 21.333 | 36.138 | 6.012 | 0.976 | 4.09% | 3.54% | Excellent |
| Time MP→TO (ms) | 0.975 | 0.933–0.990 | 198.992 | 69.333 | 418.674 | 20.462 | 3.229 | 1.62% | 1.51% | Excellent |
| Stride Angle (°) | 0.968 | 0.921–0.987 | 1.744 | 2.967 | 0.489 | 0.699 | 0.125 | 7.17% | 7.32% | Excellent |
| Total Shock (g) | 0.940 | 0.858–0.976 | 5392.667 | 2917.333 | 572747.629 | 756.801 | 184.700 | 3.43% | 3.42% | Excellent |
| Shock (g) | 0.911 | 0.792–0.963 | 15.027 | 5.533 | 2.885 | 1.699 | 0.508 | 3.38% | 3.36% | Excellent |
| Impact Gs (g) | 0.830 | 0.619–0.929 | 12.734 | 5.333 | 2.555 | 1.599 | 0.658 | 5.17% | 5.27% | Good |
| Braking Gs (g) | 0.936 | 0.846–0.974 | 7.615 | 7.333 | 3.486 | 1.867 | 0.473 | 6.22% | 6.08% | Excellent |
| Peak Vertical GRF (BW) | 0.972 | 0.908–0.990 | 3.292 | 1.500 | 0.175 | 0.418 | 0.070 | 2.12% | 1.86% | Excellent |
| Vertical GRF Rate (N·kg⁻¹·s⁻¹) | 0.981 | 0.928–0.994 | 38.615 | 14.367 | 12.149 | 3.486 | 0.476 | 1.23% | 1.05% | Excellent |
| Horizontal GRF Rate (N·kg⁻¹·s⁻¹) | 0.950 | 0.423–0.988 | 6.795 | 6.467 | 2.084 | 1.444 | 0.322 | 4.73% | 3.00% | Excellent |
| Leg Spring Stiffness (kN/m) | 0.957 | 0.850–0.985 | 9.062 | 7.967 | 2.890 | 1.700 | 0.352 | 3.89% | 3.35% | Excellent |
| Swing Force Rate (N·kg⁻¹·s⁻¹) | 0.955 | 0.801–0.985 | 1.352 | 1.133 | 0.072 | 0.269 | 0.057 | 4.24% | 3.46% | Excellent |
| Pronation Excursion (°) | 0.156 | -0.294–0.551 | -18.224 | 149.033 | 541.403 | 23.268 | 21.378 | 117.30% | 117.32% | Poor |
| Max Pronation Velocity (°/s) | 0.971 | 0.929–0.988 | 722.992 | 855.333 | 34571.690 | 185.935 | 31.691 | 4.38% | 4.47% | Excellent |
| Sacral medio-lateral GS (g) | 0.921 | 0.815–0.968 | 3.239 | 5.867 | 2.376 | 1.541 | 0.433 | 13.36% | 13.26% | Excellent |
| Sacral Vertical Oscillation (cm) | 0.987 | 0.967–0.995 | 6.653 | 5.133 | 1.884 | 1.372 | 0.157 | 2.37% | 2.43% | Excellent |
| Sacral Braking GS (g) | 0.971 | 0.927–0.989 | 3.426 | 8.433 | 3.948 | 1.987 | 0.336 | 9.80% | 9.36% | Excellent |
| Sacral Impact GS (g) | 0.950 | 0.876–0.980 | 4.893 | 7.300 | 4.563 | 2.136 | 0.479 | 9.79% | 9.44% | Excellent |
| **Note.** Test–retest reliability was assessed in a subgroup of 20 recreational runners during treadmill running at 85% of maximal aerobic speed (MAS). For each participant and session, values were averaged across the three repeated runs performed at the same speed before calculating reliability indices. Mean, range, variance, and SD values correspond to the participant-level test–retest data from this reliability subgroup and should not be interpreted as values from the full study sample. Relative reliability was assessed using the intraclass correlation coefficient (ICC), based on a two-way mixed-effects model, absolute agreement, and single measurements. ICC values were interpreted as follows: < .50 = poor, .50–.75 = moderate, .75–.90 = good, and > .90 = excellent reliability [22]. SEM was calculated as SD × √(1 − ICC), SEM% as (SEM / Mean) × 100, and CV% as (within-subject SD / Mean) × 100, with within-subject SD estimated as the standard deviation of the paired test–retest differences divided by √2. Lower SEM% and CV% values indicate lower absolute measurement error. SD = standard deviation; 95% CI = 95% confidence interval; Category = qualitative classification of reliability. | | | | | | | | | | |

**Table S4 |** Abbreviations (alphabetical)

| **Abbreviation / symbol** | **Meaning** |
| --- | --- |
| 1% | Treadmill incline (inclination grade). |
| 3D | Three-dimensional. |
| · (e.g., km·h⁻¹) | “Per” notation in compound units. |
| ⁻¹ | Inverse unit (“per”; e.g., h⁻¹ = per hour). |
| ×BW | Normalized to body weight (expressed in multiples of body weight). |
| ≥ | Greater than or equal to. |
| α | Alpha level (significance threshold; Type I error rate). |
| ° | Degrees. |
| °C | Degrees Celsius. |
| °·s⁻¹ | Degrees per second. |
| BG | Braking Gs. |
| BW | Body weight. |
| CER UVic-UCC | Research Ethics Committee of the Universitat de Vic – Universitat Central de Catalunya |
| cm | Centimeter. |
| CT | Contact time. |
| CV% | Coefficient of variation (%). |
| FDR | False discovery rate. |
| FT | Flight time. |
| g / Gs (unit) | Acceleration expressed relative to gravitational acceleration (“g”); Gs denotes values expressed in g units. |
| G*Power | G*Power statistical power-analysis software. |
| GRF | Ground reaction force. |
| HGRF_R | Horizontal ground reaction force rate. |
| Hz | Hertz (samples per second). |
| ICC | Intraclass correlation coefficient. |
| IMU / IMUs | Inertial measurement unit(s). |
| kg | Kilogram. |
| km·h⁻¹ | Kilometers per hour. |
| kN·m⁻¹ | Kilonewtons per meter. |
| LL | Lower limbs. |
| LSS | Leg spring stiffness. |
| m | Meter. |
| m/s | Meters per second. |
| MAS | Maximal aerobic speed. |
| min | Minutes. |
| MPV | Maximum pronation velocity. |
| ms | Milliseconds. |
| n | Sample size. |
| N·kg⁻¹·s⁻¹ | Newtons per kilogram per second. |
| p | p-value. |
| p_FDR | False-discovery-rate-adjusted p-value. |
| PE | Pronation excursion. |
| PVGRF (×BW) | Peak vertical ground reaction force (normalized to body weight). |
| R² | Coefficient of determination. |
| RE | Running economy. |
| RS | Running speed. |
| S_BG | Sacral braking Gs. |
| S_IG | Sacral impact Gs. |
| S_MLG | Sacral medio-lateral Gs. |
| S_VOSC | Sacral vertical oscillation. |
| SA | Stride angle. |
| SD | Standard deviation. |
| SEM | Standard error of measurement. |
| SEM% | Standard error of measurement expressed as a percentage of the mean. |
| SF_R | Swing force rate. |
| SH | Shock. |
| SL | Stride length. |
| SPSS | IBM SPSS Statistics software. |
| SR | Step rate. |
| steps·min⁻¹ | Steps per minute. |
| T | Time outcome derived from the RunScribe™ system (MP→TO). |
| TM | Treadmill. |
| TS | Total shock. |
| VGRF_R | Vertical ground reaction force rate. |

**Meaning of “65% of MAS”, “85% of MAS”, and “100% of MAS”**

These values indicate relative treadmill running speeds, expressed as a percentage of each participant’s individual Maximal Aerobic Speed (MAS):

- 65% MAS = 0.65 × MAS
- 85% MAS = 0.85 × MAS
- 100% MAS = 1.00 × MAS
